# Identification of rs671, a common variant of *ALDH2*, as a gout susceptibility locus

**DOI:** 10.1038/srep25360

**Published:** 2016-05-16

**Authors:** Masayuki Sakiyama, Hirotaka Matsuo, Hirofumi Nakaoka, Ken Yamamoto, Akiyoshi Nakayama, Takahiro Nakamura, Sayo Kawai, Rieko Okada, Hiroshi Ooyama, Toru Shimizu, Nariyoshi Shinomiya

**Affiliations:** 1 Department of Integrative Physiology and Bio-Nano Medicine, National Defense Medical College, 3-2 Namiki, Tokorozawa, Saitama 359-8513, Japan; 2 Department of Dermatology, National Defense Medical College, 3-2 Namiki, Tokorozawa, Saitama 359-8513, Japan; 3 Division of Human Genetics, Department of Integrated Genetics, National Institute of Genetics, 1111 Yata, Mishima, Shizuoka 411-0801, Japan; 4 Department of Medical Chemistry, Kurume University School of Medicine, 67 Asahi-machi, Kurume, Fukuoka 830-0011, Japan; 5 Laboratory for Mathematics, National Defense Medical College, 3-2 Namiki, Tokorozawa, Saitama 359-8513, Japan; 6 Department of Preventive Medicine, Nagoya University Graduate School of Medicine, 65 Tsurumai-cho, Showa-ku, Nagoya, Aichi 466-8550, Japan; 7 Ryougoku East Gate Clinic, 3-21-1 Ryougoku, Sumida-ku, Tokyo 130-0026, Japan; 8 Midorigaoka Hospital, 3-13-1 Makami-cho, Takatsuki, Osaka 569-1121, Japan; 9 Kyoto Industrial Health Association, 67 Kitatsuboi-cho, Nishinokyo, Nakagyo-ku, Kyoto 604-8472, Japan

## Abstract

Gout is a common disease resulting from hyperuricemia. Recently, a genome-wide association study identified an association between gout and a single nucleotide polymorphism (SNP) rs2188380, located on an intergenic region between *MYL2* and *CUX2* on chromosome 12. However, other genes around rs2188380 could possibly be gout susceptibility genes. Therefore, we performed a fine-mapping study of the *MYL2-CUX2* region. From 8,595 SNPs in the *MYL2-CUX2* region, 9 tag SNPs were selected, and genotyping of 1,048 male gout patients and 1,334 male controls was performed by TaqMan method. Eight SNPs showed significant associations with gout after Bonferroni correction. rs671 (Glu504Lys) of *ALDH2* had the most significant association with gout (*P* = 1.7 × 10^−18^, odds ratio = 0.53). After adjustment for rs671, the other 8 SNPs no longer showed a significant association with gout, while the significant association of rs671 remained. rs671 has been reportedly associated with alcohol drinking behavior, and it is well-known that alcohol drinking elevates serum uric acid levels. These data suggest that rs671, a common functional SNP of *ALDH2*, is a genuine gout-associated SNP in the *MYL2-CUX2* locus and that “A” allele (Lys) of rs671 plays a protective role in the development of gout.

Gout is a common disease resulting from hyperuricemia, and causes acute arthritis. Previous genetic and functional analyses revealed that *ABCG2* dysfunctional variants caused gout^1^,^2^,^3^ due to decreased urate excretion in gut^4^ and kidney^5^. Genome-wide association studies (GWASs) of gout also showed genome-wide significant associations with *ABCG2* and *GLUT9*^6^,^7^,^8^. Recently, we revealed for the first time that the following 3 loci were associated with gout at the genome-wide significance level: rs1260326 of *GCKR*, rs4073582 of *CNIH-2* and rs2188380 of *MYL2-CUX2*^8^. Among them, 2 SNPs are located in gene regions: rs1260326 is a nonsynonymous single nucleotide polymorphism (SNP) (Leu446Pro) of *GCKR*, and rs4073582 is an intronic SNP of *CNIH-2*. On the other hand, rs2188380 is located on an intergenic region between *MYL2* and *CUX2*^8^. Additionally, we detected many SNPs showing significant associations with gout across the chromosome 12q24 region which were in strong linkage disequilibrium (LD) with rs2188380. *MYL2* encodes a regulatory light chain associated with cardiac myosin β (or slow) heavy chain, and an association between *MYL2* variant and high-density lipoprotein cholesterol metabolism was previously reported^9^. *CUX2* regulates cell-cycle progression^10^ and plays important roles in neural progenitor development in the central nervous system^10^,^11^. An association between *CUX2* and type 1 diabetes has also been reported^12^. However, there is a possibility that the other genes around rs2188380 of *MYL2-CUX2* can be gout susceptibility genes. Therefore, we performed fine-mapping of the *MYL2-CUX2* region and a further association analysis of gout.

## Results

**Figure 1:**
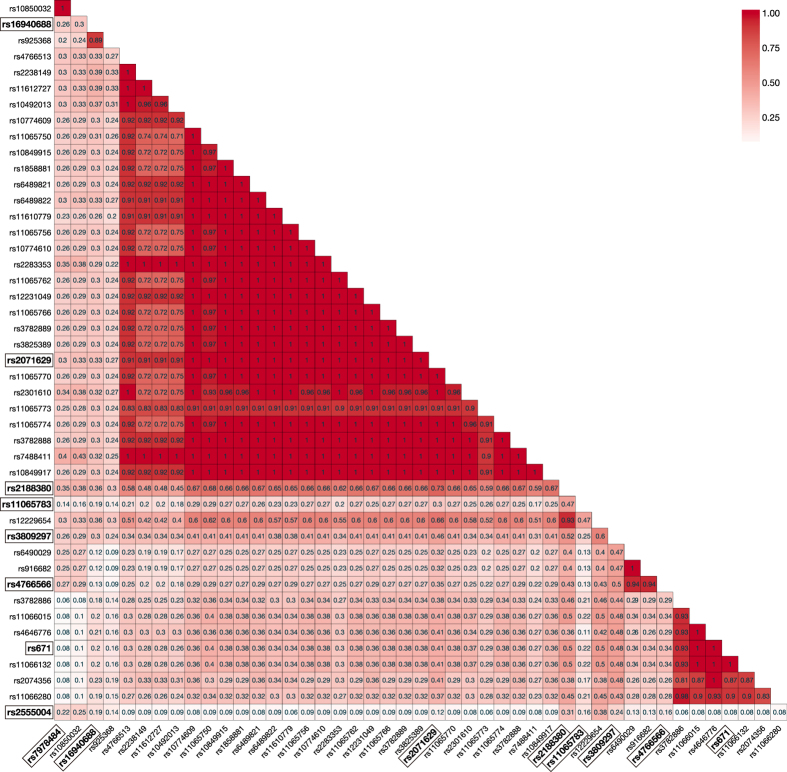
Linkage disequilibrium heat map of 46 SNPs. We examined the linkage disequilibrium (LD) between each pair of 46 SNPs and searched the SNPs that were tagging other SNPs with strong LD (*r*^*2*^ ≥ 0.8). The 9 tag SNPs (rs7978484, rs16940688, rs2071629, rs2188380, rs11065783, rs3809297, rs4766566, rs671 and rs2555004), which are shown in bold and boxes, were selected for association analysis.

From 8,595 SNPs in the *MYL2-CUX2* region within 10 Mb across rs2188380, 45 SNPs in LD (*r*^*2*^ ≥ 0.3) with rs2188380 were selected (Supplementary Figure S1). Among these 45 SNPs and rs2188380, 9 tag SNPs were selected for association analysis (Fig. 1 and Supplementary Table S1). Genotyping results of the 9 tag SNPs for 1,048 gout patients and 1,334 controls were shown in Table 1. The call rates for the 9 SNPs were more than 95.0%. All the SNPs in the control group were in Hardy-Weinberg equilibrium (*P* > 0.05). Except for rs2555004, the other 8 SNPs showed significant associations at *P* < 5.6 × 10^−3^ (=0.05/9) with the Bonferroni correction, and rs671 (Glu504Lys) of aldehyde dehydrogenase 2 (*ALDH2*) had the most significant association with gout (*P* = 1.7 × 10^−18^; odds ratio [OR] = 0.53; 95% confidence interval [CI]: 0.46–0.61, Table 1 and Supplementary Figure S2A).

Next, the multivariate logistic regression analyses were performed to evaluate whether there was an additional association signal after the adjustment for the most significantly associated SNP rs671. We set the significance threshold as α = 6.3 × 10^−3^ (=0.05/8) with the Bonferroni correction. While the significant association of rs671 remained, the other 8 SNPs no longer showed a significant association with gout after the adjustment for rs671 (Table 2 and Supplementary Figure S2B). Among rs671 and 6 tagged SNPs (rs3782886, rs11066015, rs4646776, rs11066132, rs2074356 and rs11066280) shown in Supplementary Table S2, rs671 is the most promising functional SNP because only rs671 is a nonsynonymous variant (Glu504Lys). rs4766566 had a nominally significant association (*P* = 0.032), but did not pass the significant threshold for multiple hypothesis testing (Table 2). Additionally, the OR for rs4766566 became closer to 1.0 after the adjustment for rs671 (from 0.59 to 0.82; Tables 1 and 2). These data suggest that rs671 (Glu504Lys) of *ALDH2* is a genuine gout-associated SNP in the *MYL2-CUX2* locus.

It is well-known that individuals with rs671 heterozygotes (A/G; Lys/Glu) have only 6.25% of enzyme activity of those with normal ALDH2 (G/G; Glu/Glu), and those with homozygotes (A/A; Lys/Lys) show almost no activity^13^. Therefore, it is expected that dominant model (G/G v.s. A/G or A/A) is the most likely genetic model for the association between rs671 and gout. Actually, the statistical significance of the association between rs671 and gout was improved by applying the dominant model (*P* = 2.9 × 10^−21^; OR = 0.44; 95% CI: 0.37–0.52) compared with the result from the allelic model (*P* = 1.7 × 10^−18^; OR = 0.53; 95% CI: 0.46–0.61; Table 1). These findings indicate that “A” allele (Lys) of rs671 plays a protective role in the development of gout.

## Discussion

In this study, among 9 tag SNPs selected from 8,595 SNPs in the *MYL2-CUX2* locus, only the association of rs671 of *ALDH2* remained significant after the adjustment for each SNP with the Bonferroni correction (Table 2). Together with the fact that rs671 (Glu504Lys) is a well-known dysfunctional SNP, we therefore concluded that rs671 is a genuine gout-associated SNP. Indeed, a previous Japanese study with 180 gout cases and 49 controls has indicated that the frequency of homozygotes (A/A; Lys/Lys) of rs671 was lower in gout patients than in controls^14^. The fine-mapping study for the associational signal on chromosome 12 identified by our GWAS reached the consistent result with the previous finding showing the association between *ALDH2* and gout^14^.

ALDH2 is a crucial enzyme in the alcohol metabolism. Alcohol is oxidized to acetaldehyde by alcohol dehydrogenase, and acetaldehyde is further metabolized to acetate by aldehyde dehydrogenase^15^, which largely depends on ALDH2. rs671 (Glu504Lys), a common missense SNP of *ALDH2* gene, severely decreases the activity of ALDH2 enzyme^13^. When acetate is metabolized to acetyl-coenzyme A, adenosine triphosphate (ATP) hydrolyzes to adenosine monophosphate (AMP) which is ultimately metabolized to uric acid. This alcohol metabolism is one of the reasons why alcohol drinking elevates serum uric acid (SUA) levels. Thus, the association between rs671 and gout is partly due to alcohol drinking.

The allele frequencies of rs671 of *ALDH2* differ among populations: the 504Lys allele (“A” allele) is common in East Asians including Japanese, but quite rare in other populations such as European and African descendants^16^. Therefore, it is reasonable that this SNP has not been detected in the previous GWASs of gout in Europeans and African Americans due to its low frequency. We showed that rs671 of *ALDH2* is an influential genetic factor for Japanese as the other 4 previously reported loci^8^ (*ABCG2*, *SLC2A9*, *GCKR* and *CNIH-2*) of gout, and further investigations in East Asian populations will be able to warrant these findings.

In summary, Glu504Lys polymorphism (rs671), a common dysfunctional SNP of *ALDH2*, is identified as a genuine gout-associated polymorphism in the *MYL2-CUX2* locus, and “A” allele (Lys) of rs671 plays a protective role in the development of gout.

## Methods

### Study participants

This study was approved by the institutional ethical committees (National Defense Medical College and Nagoya University), and all procedures involved in this study were performed in accordance with the Declaration of Helsinki with written informed consent from each subject.

1,048 gout cases were assigned from the Japanese male outpatients at the gout clinics of Kyoto Industrial Health Association (Kyoto, Japan), or Ryougoku East Gate Clinic (Tokyo, Japan). All patients were clinically diagnosed as primary gout according to the criteria established by the American College of Rheumatology^17^. Patients with inherited metabolism disorders including Lesch–Nyhan syndrome were excluded. For the control group, 1,334 Japanese males with normal SUA (≤7.0 mg/dl) and without a history of gout were collected from the participants in the Shizuoka area in the Japan Multi-Institutional Collaborative Cohort Study (J-MICC Study)^18^,^19^. The details of participants in this study are shown in Supplementary Table S3.

### Selection of SNPs

First, 8,595 SNPs within 10 Mb across rs2188380 were selected using HapMap phase III JPT samples (http://hapmap.ncbi.nlm.nih.gov/)^20^; then, the pairwise LD was calculated between rs2188380 and the 8,595 SNPs (Supplementary Figure S1). After 8,550 SNPs in weak LD were excluded, the other 45 SNPs showing moderate to strong LD (*r*^*2*^ ≥ 0.3) with rs2188380 remained. Next, we examined the LD between each pair of these 46 SNPs (Fig. 1), and searched for the SNPs that were tagging other SNPs with strong LD (*r*^*2*^ ≥ 0.8). Finally, in addition to rs2188380, we selected 8 SNPs (rs7978484, rs16940688, rs2071629, rs11065783, rs3809297, rs4766566, rs671 and rs2555004) for association analysis (Supplementary Table S1).

### Genetic analysis

Genomic DNA was extracted from whole peripheral blood cells^21^. Genotyping of the 8 SNPs was performed by the TaqMan method (Life Technologies Corporation, Carlsbad, CA USA) with a LightCycler 480 (Roche Diagnostics, Mannheim, Germany)^22^. To confirm their genotypes, DNA sequencing analyses were performed with the primers shown in Supplementary Table S4. Direct sequencing was performed with a 3130xl Genetic Analyzer (Life Technologies Corporation)^22^. The deviation from Hardy-Weinberg equilibrium in control samples was evaluated by chi-square test using the software R (version 3.1.1) (http://www.r-project.org/).

### Statistical analyses

The associations between SNPs and gout were examined with logistic regression analyses. For the robustness of the statistical test, random re-sampling methods with computer simulation are often applied^23^,^24^. In this study, the permutation test^24^ was used for random re-sampling in a case-control study with replacement for 1,000,000 times, and the robustness of statistics was confirmed. All the logistic regression analyses and chi-square tests were performed with SPSS v.22.0J (IBM Japan Inc., Tokyo, Japan) and the software R (version 3.1.1) (http://www.r-project.org/). We examined the pairwise LD using PLINK v1.07^25^.

## Additional Information

**How to cite this article**: Sakiyama, M. *et al.* Identification of rs671, a common variant of *ALDH2*, as a gout susceptibility locus. *Sci. Rep.* **6**, 25360; doi: 10.1038/srep25360 (2016).

## Supplementary Material

Supplementary Information

## Figures and Tables

**Table 1:** Association analysis of gout.

SNP*	Position†	Gene	*r*^*2*^‡	*D′*‡	A1/A2§	MAF		*P* value¶		Reciprocal OR (95% CI)
						Cases	Controls		OR (95% CI)	
rs2188380	111386127	*MYL2-CUX2*	–	–	T/C	0.14	0.22	7.1 × 10^−12^	0.58 (0.50–0.68)	1.72 (1.47–2.00)
rs7978484	109738076	*FOXN4*	0.35	0.65	G/A	0.14	0.17	4.8 × 10^−3^	0.79 (0.67–0.93)	1.26 (1.07–1.49)
rs16940688	110360321	*TCHP-GIT2*	0.36	0.85	G/A	0.07	0.13	6.2 × 10^−9^	0.55 (0.45–0.67)	1.82 (1.49–2.23)
rs2071629	111351186	*MYL2*	0.73	0.90	G/A	0.17	0.25	3.7 × 10^−11^	0.60 (0.52–0.70)	1.65 (1.42–1.92)
rs11065783	111396249	*MYL2-CUX2*	0.47	1.00	A/G	0.25	0.31	2.8 × 10^−5^	0.76 (0.66–0.86)	1.32 (1.16–1.51)
rs3809297	111609727	*CUX2*	0.52	0.79	G/T	0.17	0.28	6.7 × 10^−16^	0.55 (0.48–0.64)	1.82 (1.57–2.10)
rs4766566	111706877	*CUX2*	0.43	1.00	T/C	0.25	0.36	1.2 × 10^−15^	0.59 (0.52–0.67)	1.69 (1.48–1.92)
rs671	112241766	*ALDH2*	0.50	0.91	G/A	0.18	0.29	1.7 × 10^−18^	0.53 (0.46–0.61)	1.88 (1.63–2.16)
rs2555004	114686645	*RBM19-TBX5*	0.31	0.82	A/G	0.21	0.20	0.22	1.09 (0.95–1.26)	0.91 (0.79–1.06)

Abbreviation: MAF = minor allele frequency; OR = odds ratio; CI = confidence interval.

^*^dbSNP rs number.

^†^SNP positions are based on NCBI human genome reference sequence Build 37.

^‡^*r*^*2*^ and *D ′* indicate the pairwise linkage disequilibrium with rs2188380.

^§^A1 is a major allele and A2 is a minor allele.

^¶^*P* values smaller than 5.6 × 10^−3^ (adjusting for 9 tests with the Bonferroni correction) are shown in bold letters.

**Table 2:** Multivariate logistic regression analysis of gout including rs671 and each of the 8 SNPs.

SNP A	SNP A		rs671	
	*P* value*	OR (95% CI)	*P* value†	OR (95% CI)
rs7978484	0.390	0.94 (0.74–1.19)	2.6 × 10^−16^	0.57 (0.46–0.70)
rs16940688	0.054	0.93 (0.78–1.10)	1.6 × 10^−11^	0.54 (0.47–0.63)
rs2071629	0.228	0.80 (0.63–1.00)	7.8 × 10^−10^	0.57 (0.49–0.67)
rs2188380	0.593	0.89 (0.73–1.08)	2.1 × 10^−7^	0.56 (0.47–0.67)
rs11065783	0.195	1.11 (0.95–1.31)	6.8 × 10^−16^	0.48 (0.41–0.58)
rs3809297	0.213	0.85 (0.66–1.10)	3.1 × 10^−5^	0.59 (0.46–0.76)
rs4766566	0.032	0.82 (0.68–0.98)	3.2 × 10^−6^	0.61 (0.50–0.75)
rs2555004	0.353	1.07 (0.93–1.24)	9.4 × 10^−19^	0.52 (0.45–0.60)

Abbreviation: OR = odds ratio; CI = confidence interval.

^*^P values of each of the 8 SNPs (SNP A) adjusted by rs671.

^†^*P* values of rs671 adjusted by SNP A. *P* values smaller than 6.3 × 10^−3^ (adjusting for 8 tests with the Bonferroni correction) are shown in bold letters.
