# Supplementary material for: Identification of rs671, a common variant of ALDH2, as a gout susceptibility locus
Source: Sci Rep. 2016 May 16;6:25360. doi: 10.1038/srep25360 (PMC4867610; doi:10.1038/srep25360)
Supplement: Supplementary Information [file srep25360-s1.doc]

SUPPLEMENTARY INFORMATION

**Identification of rs671, a common variant of *ALDH2*,**

**as a gout susceptibility locus**

Masayuki Sakiyama, Hirotaka Matsuo*, Hirofumi Nakaoka, Ken Yamamoto, Akiyoshi Nakayama, Takahiro Nakamura, Sayo Kawai, Rieko Okada,

Hiroshi Ooyama, Toru Shimizu & Nariyoshi Shinomiya

*Corresponding author. E-mail: hmatsuo@ndmc.ac.jp

Supplementary Figure S1 | Method of SNP selection for fine-mapping

Supplementary Figure S2 | Regional plots of the 9 tag SNPs in the *MYL2-CUX2* region

Supplementary Table S1 | Selected 9 SNPs in moderate to strong linkage disequilibrium with rs2188380

Supplementary Table S2 | rs671 and 6 tagged SNPs

Supplementary Table S3 | Clinical characteristics of participants for association analysis

Supplementary Table S4 | Primers for direct sequencing of 8 SNPs

**
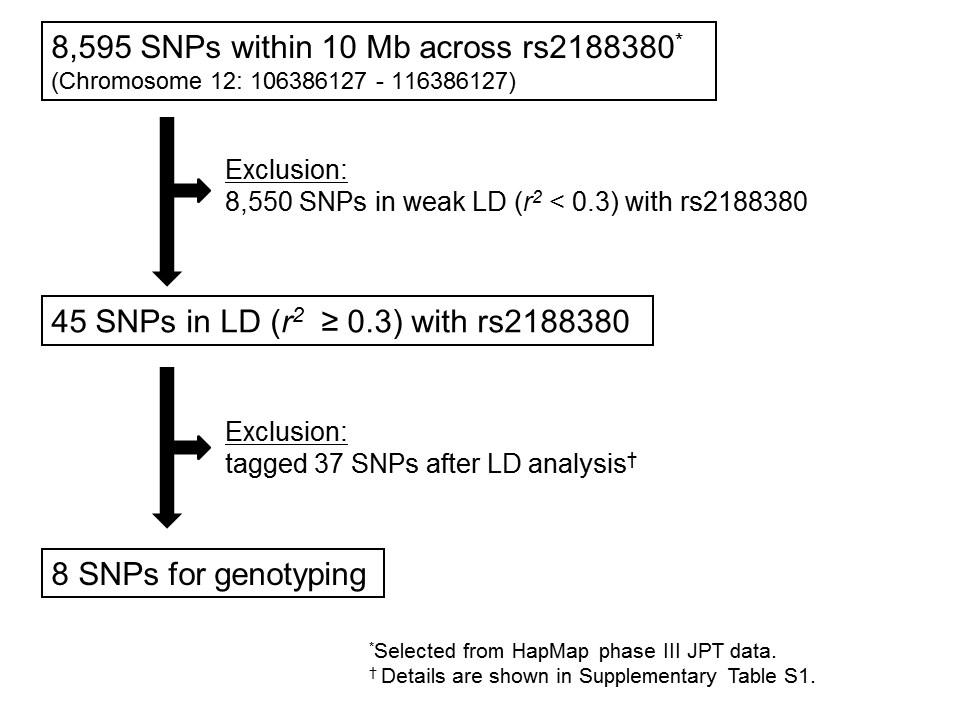
**

**Supplementary Figure S1 | Method of SNP selection** **for fine-mapping.** At first, 8,595 SNPs within 10 Mb across rs2188380 were selected using HapMap phase III JPT samples. After the pairwise LD was calculated between rs2188380 and 8,595 SNPs, 45 SNPs showing moderate to strong LD (*r^2^* ≥ 0.3) with rs2188380 were selected while the other 8,550 SNPs in weak LD were excluded. Next, we examined the LD between each pair of these 46 SNPs (45 SNPs and rs2188380), and excluded 37 SNPs which were tagging other SNPs with strong LD (*r^2^* ≥ 0.8). Finally, in addition to rs2188380, we selected 8 SNPs (rs7978484, rs16940688, rs2071629, rs11065783, rs3809297, rs4766566, rs671 and rs2555004) for genotyping.


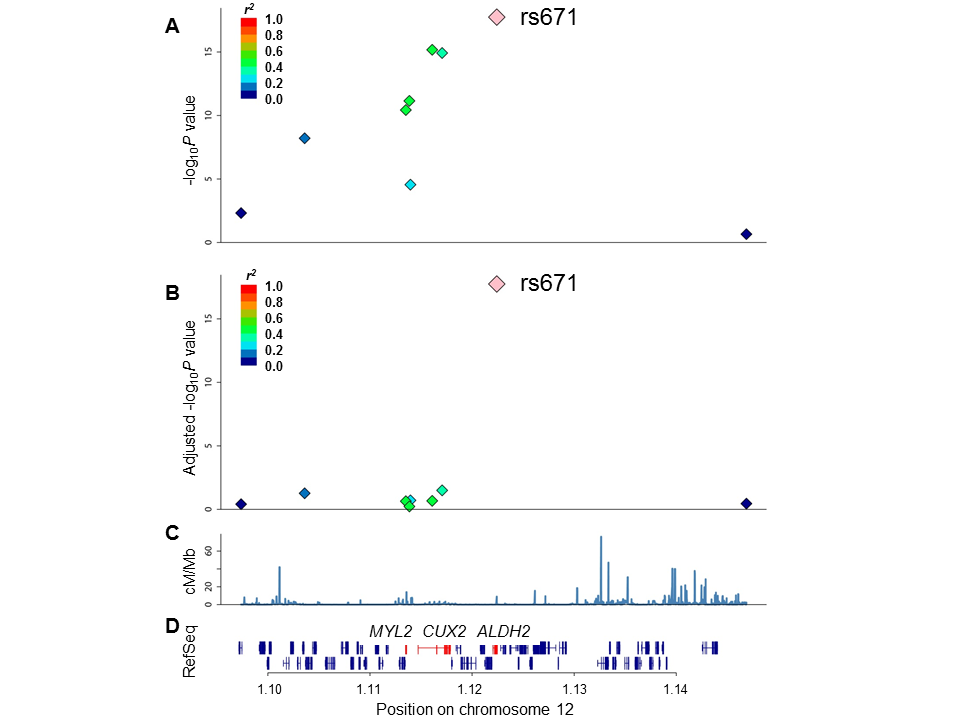


**Supplementary Figure S2 | Regional plots of the 9 tag SNPs in the *MYL2-CUX2* region.** (A) Vertical axis shows -log_10_ *P* values for the test of SNP association with gout. 8 SNPs showed significant associations (*α =* 5.6 × 10^-3^). Among the 8 SNPs, rs671 (Glu504Lys) of *ALDH2* had the highest association signal with gout. (B) Regional plot after the adjustment for rs671. The 8 SNPs other than rs671 no longer showed a significant association with gout. For panels A and B, SNP showing the lowest *P* value is depicted as a pink diamond. Other SNPs are color-coded according to the extent of linkage disequilibrium (measured in *r^2^*) with SNP showing the lowest *P* value. (C) Recombination rates (centimorgans per Mb) estimated from HapMap Phase III data are plotted. (D) RefSeq genes. Genomic coordinates are based on Genomic Reference Consortium GRCh37.

Supplementary Table S1 | Selected 9 SNPs in moderate to strong linkage disequilibrium with rs2188380

| SNP^*^ | Position^†^ | Gene | Tagged SNP with *r^2^* >0.8 |
| --- | --- | --- | --- |
|
| rs2188380 | 111386127 | *MYL2-CUX2* | rs12229654 |
| rs7978484 | 109738076 | *FOXN4* | rs10850032 |
| rs16940688 | 110360321 | *TCHP-GIT2* | rs925368 |
| rs2071629 | 111351186 | *MYL2* | rs11065750, rs12231049, rs3782888, rs10849917, rs10774609, rs6489821, rs10849915, rs1858881, rs11065756, rs10774610, rs11065762, rs11065766, rs3782889, rs3825389, rs11065770, rs11065774, rs6489822, rs11610779, rs2301610, rs2283353, rs7488411, rs11065773, rs4766513, rs2238149, rs11612727, rs10492013 |
| rs11065783 | 111396249 | *MYL2-CUX2* | - |
| rs3809297 | 111609727 | *CUX2* | - |
| rs4766566 | 111706877 | *CUX2* | rs6490029, rs916682 |
| rs671 | 112241766 | *ALDH2* | rs3782886, rs11066015, rs4646776, rs11066132, rs2074356, rs11066280 |
| rs2555004 | 114686645 | *RBM19-TBX5* | - |

^*^dbSNP rs number.

^†^SNP positions are based on NCBI human genome reference sequence Build 37.

Supplementary Table S2 | rs671 and 6 tagged SNPs

| SNP^*^ | Position^†^ | Gene | Functional consequence | *r^2^* with rs671 |
| --- | --- | --- | --- | --- |
| rs671 | 112241766 | *ALDH2* | missense | - |
| rs3782886 | 112110489 | *BRAP* | synonymous | 0.93 |
| rs11066015 | 112168009 | *ACAD10* | intron | 1.00 |
| rs4646776 | 112230019 | *ALDH2* | intron | 1.00 |
| rs11066132 | 112468206 | *NAA25* | intron | 1.00 |
| rs2074356 | 112645401 | *HECTD4* | intron | 0.87 |
| rs11066280 | 112817783 | *HECTD4* | intron | 0.90 |

^*^dbSNP rs number.

^†^SNP positions are based on NCBI human genome reference sequence Build 37.

Supplementary Table S3 | Clinical characteristics of participants for association analysis

|  | Cases | Controls |
| --- | --- | --- |
| Number | 1,048 | 1,334 |
| Age (year) | 44.9 ± 11.4 | 52.4 ± 8.6 |
| Body-mass index (kg/m^2^) | 25.0 ± 3.5 | 23.2 ± 2.7 |

Plus-minus values are means ± SD.

Supplementary Table S4 | Primers for direct sequencing of 8 SNPs

| SNP^*^ | Gene | Forward primers | Reverse primers |
| --- | --- | --- | --- |
| rs7978484 | *FOXN4* | 5’-GTCCCTGGTTCAAATCCC-3’ | 5’-AGTATGGAGAAGTAGACCCCG-3’ |
| rs16940688 | *TCHP-GIT2* | 5’-AAGTCTCTGCTCCACACCTC-3’ | 5’-TAGTAGAGATGGGGCTTTGC-3’ |
| rs2071629 | *MYL2* | 5’-GATGGGGAACTGAGACTAAGAG-3’ | 5’-CCCACTCACTAATCAGCCTTC-3’ |
| rs11065783 | *MYL2-CUX2* | 5’-ACTCCCTCATCCATTCTTCC-3’ | 5’-CTTTCTCTCTCCTCAGTGGC-3’ |
| rs3809297 | *CUX2* | 5’-GCTCCCTGGTGAAAGAATG-3’ | 5’-GCACACACACACACAAGC-3’ |
| rs4766566 | *CUX2* | 5’-AAATGACACTGGAGGATGACC-3’ | 5’-CTCCAATAACCTTTCCTCCC-3’ |
| rs671 | *ALDH2* | 5’-AGGAGAATCTCTTGAACCCC-3’ | 5’-CTCTCTTGTCACTTCTCAGGC-3’ |
| rs2555004 | *RBM19-TBX5* | 5’-GAATGCCAAGTGCCTGAG-3’ | 5’-GGGGAGGAAGAAGAATGC-3’ |

^*^dbSNP rs number.
